# Cellular iron storage and trafficking are affected by GTN stimulation in primary glial and meningeal cell culture

**DOI:** 10.3906/biy-2009-1

**Published:** 2021-02-09

**Authors:** Latife Arzu ARAL*, Mehmet Ali ERGÜN, Hayrunnisa BOLAY

**Affiliations:** 1 Department of Immunology, Faculty of Medicine, İzmir Demokrasi University, İzmir Turkey; 2 Department of Medical Genetics, Faculty of Medicine, Gazi University, Ankara Turkey; 3 Department of Neurology, Faculty of Medicine, Gazi University, Ankara Turkey

**Keywords:** Migraine, microglia, astrocyte, meningeal cell, iron, ferritin, MMP-9, CGRPR

## Abstract

A well-balanced intracellular iron trafficking in glial cells plays a role in homeostatic processes. Elevated intracellular iron triggers oxidative stress and cell damage in many neurological disorders, including migraine. This study aimed to investigate the effects of glyceryl trinitrate (GTN), on cellular iron homeostasis, matrixmetalloproteinase (MMP)-9, and calcitonin gene related peptide (CGRP) receptor (CRLR/CGRPR1) production in microglia, astrocyte, and meningeal cell cultures. Primary glial and meningeal cells in culture were exposed to GTN for 24 h. Messenger RNA expression was assessed using qPCR. Iron accumulation was visualized via modified Perl’s histochemistry. MMP-9 levels in cell culture supernatants were measured using ELISA. Ferritin and CRLR/CGRPR1 proteins were visualized via immunofluorescence staining. Nitric oxide production increased significantly with GTN in meningeal and glial cells. GTN significantly increased the expression of the storage protein ferritin for all three cell types, but ferritin-L for meningeal cells and microglia. Iron trafficking associated with the efflux protein ferroportin and influx protein divalent metal transporter (DMT)1 was affected differently in all three cell types. MMP-9 expression was increased in astrocytes. GTN stimulation increased both CRLR/CGRPR1 expression, and immunostaining was apparent in microglia and meningeal cells. This study showed for the first time that GTN modulates intracellular iron trafficking regulated by storage and transport proteins expressed in meningeal cells and glia. CRLR/CGRPR1 expression might be related to altered iron homeostasis and they both may stimulate nociceptive pathways activated in migraine. These molecules expressed differently in glial and meningeal cells in response to GTN may bring not only new targets forward in treatment but also prevention in migraine.

## 1. Introduction

Accumulating cellular iron was found to differ in chronic and episodic migraine patients and was associated with the duration of pain; however, no iron accumulation was shown in headache triggered by medication or stress (Tepper et al., 2012). Free radical damage associated with hyperemia that develops during recurrent attacks was also found related with iron accumulation in brain areas activated during migraine (Goadsby 2012). Cellular accumulation of iron disrupted normal antinociceptive function by various ways and contributed to pain formation (Kruit et al., 2009). 

Glial cells, which control inflammation in homeostatic as well as neurodegenerative processes, are the key players maintaining iron in central nervous system (CNS). Microglia prevent excessive accumulation of iron by storing it in different forms (Kaur and Ling, 2009), while astrocytes regulate iron metabolism via molecules involved in iron transport and storage (Jeong and David, 2003). Matrix metalloproteinase (MMP)-9 produced by glial cells mediates the pain pathways by recruiting activated immune cells to the scene of inflammation and increasing blood-brain barrier (BBB) permeability by disrupting the endothelial cell basal laminae (Gursoy-Ozdemir et al., 2004, Gawlak et al, 2009). Studies conducted in recent years reveal that glial cells contribute to migraine pathogenesis by changing the BBB permeability (Ricci et al., 2009). A relationship between MMP-9 and cellular iron homeostasis in glial cells in migraine pathophysiology has not yet been disclosed.

Owing to its ability to gain and lose electrons easily, iron is considered an important part of metabolism. In addition to BBB, multiple control systems within the brain tissue regulate cellular iron at sufficient levels to maintain the normal functions of nervous system (Mills et al., 2010). Proteins that take iron into the cell are called influx proteins such as divalent metal transporter (DMT)1, Zip14, and transferrin receptor (TfR)1. Ferroportin (Fpn) and ceruloplasmin (Cp) are the primary efflux proteins transporting iron out of the cell (Oshiro et al., 2011). It is important that these regulatory systems work properly to protect cells from both deprivation and deposition of iron.

Ferritin, the major cellular storage form of iron, consists of subunits defined as heavy (-H) and light chain (-L). Ferritin-H is responsible for the rapid oxidation of iron in the cell (Levi et al., 1988), whereas ferritin-L is responsible for protein stability and the mineralization of iron (Santambrogio et al., 1996). Ferritin, by storing iron in the cell, makes it accessible in conditions such as myelin and enzyme synthesis, and has a protective effect in the development of free iron-mediated oxidative damage. Inflammation alters the cellular traffic of iron, and disrupted iron homeostasis, which is affected as a vicious circle, induces inflammation (Zarruk et al., 2015).

Different pathways and cells are known to play a role in the pathogenesis of migraine. Neurogenic inflammation theory, which is considered an alternative to vascular theory, is characterized by release of neuropeptides such as substance P and calcitonin gene-related peptide (CGRP). This results in plasma protein extravasation and vasodilation (Reuter et al., 2001). Blocking CGRP and its receptor components is an important target in treatment by suppressing inflammation. Besides glial cells, meningeal cells are also key players in inflammatory pathways activated during migraine (Bolay et al., 2004). Located in dural membranes, meningeal cells act on iron homeostasis, and their crosstalk with glial cells might help illuminate some unanswered questions in migraine pathophysiology. 

Glyceryletrinitrate (GTN), a NO donor known with its ability to trigger headache in migrainueurs, stimulates inflammation in the rat dura mater and is used as an approved tool to induce migraine in animal studies. In this study, the effects of GTN stimulation on cellular iron homeostasis were evaluated in primary microglia, astrocyte, and meningeal cell cultures, individually. 

## 2. Materials and methods 

**Figure 1:**
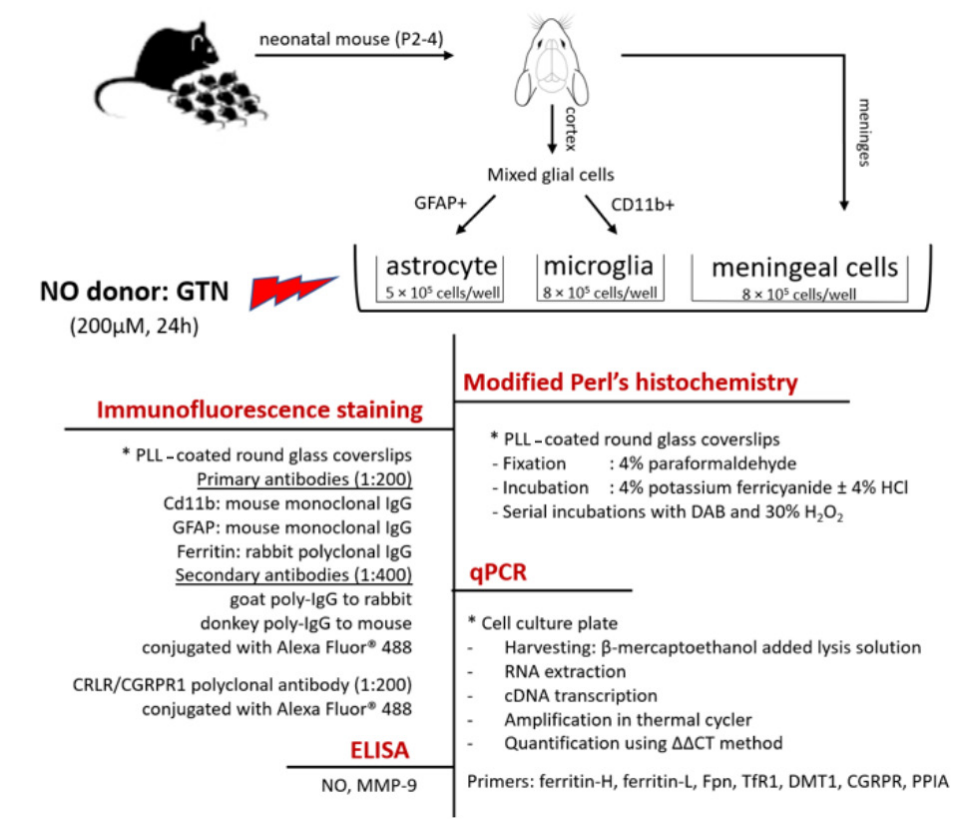
Summary of the research design.

This study has been approved by Gazi University Ethical Committee for Research Animals on 26.12.2014 with the number 66332047-604.01.02. Research design was summarized in Figure 1. 

### 2.1. GTN induction in primary cell culture and cell viability 

Glial cell cultures were prepared as described previously (Jeong and David, 2003; Saura et al., 2003). Briefly, the neonatal mouse (P2-4) cerebral cortex was dissociated and plated in poly-L-lysine (PLL)-coated tissue-culture flasks. Ten days later, the confluent cells were treated with mild trypsinization that detaches a layer of loosely adherent cells, which are mainly astrocytes, and leaves behind firmly adherent cells, which constitute >90% of microglia. Afterwards, cells were cultured for 5 days in DMEM/F12 culture medium (ThermoFisher, USA; Cat No: 11320), containing 10% FBS, 1% penicillin/streptomycin, and 1% vitamins, mixed (1:1) with glial cell-conditioned media before stimulation. For astrocytes, another group of confluent cells were left at 37 °C overnight on a horizontal shaker (200 rpm) closed with paraffin, and then the floating cells were discarded. After a mild trypsinization, cells were replated at 1/3 density in DMEM culture medium (ThermoFisher, USA; Cat No: 11995) containing 10% FBS, 1% penicillin/streptomycin, and 1% vitamins. Cells were ready to use after a 7-day mitotic inhibitor cytosine arabinoside (10 IM) treatment to remove proliferating meningeal cells. Purity of astrocyte and microglia cultures was assessed by immunofluorescence labeling for GFAP and CD11b, respectively. Purified cells were plated at a density of 8 × 10^5^ cells/well for microglia, and 5 × 10^5^ cells/well for astrocytes, 2 days before the start of the experiment. 

The meninges removed from the cortex tissue used for glial cell culture were taken into a separate petri dish for meningeal cell culture. After dividing into small pieces, tissues were trypsinized (trypsin 0.25%, catalog no: 25050-014; Gibco, Life Tech., UK), and centrifugation and filtration steps were carried out. Suspended cells were transferred to 25 cm2 sterile cell culture flasks coated with PLL (Serva Electrophoresis, Germany). Stimulations were performed simultaneously with glial cells.

The most appropriate concentration and duration for GTN stimulation was determined as cell viability using the commercial kit based on the measurement of metabolic activity of 2,3-Bis (2-methoxy-4-nitro-5-sulfophenyl)-2H-tetrazolium (XTT) as tetrazolium salt (XTT Cell Proliferation Kit, Biological Industries, Israel). This is a calorimetric method, measuring the absorbance at 450/620 nm, at 6, 12, 24, and 48 h of incubation at 37 °C. Cytotoxicity rate was accepted as >50% in culture.

### 2.2. Perl’s histochemistry 

Cellular iron accumulation was shown using modified Perl’s histochemistry as previously described in the literature (Smith et al., 1997). Briefly, cells were plated to PLL-coated glass coverslips overnight and stimulated with GTN. The coverslips were then fixed with 4% paraformaldehyde for 15 min in the wells. After washing with PBS, the coverslips were incubated in 4% potassium ferricyanide, first without, then with 4% HCl for 1 h, individually. Cells storing iron became visible with serial incubations with diaminobenzidin (DAB) (Amresco, USA) and 30% H_2_O_2_. The stained coverslips were fixed before visualizing under light microscope. 

### 2.3. Quantitative real-time polymerase chain reaction (q-PCR)

After incubation with GTN, cells were harvested using β-mercaptoethanol-added lysis solution and stored at –80 °C until RNA extraction (n = 3 per group, duplicated, pooled from 6 animals for each cell type). Total RNA was extracted using RNeasy Mini Kit (Qiagen, USA) following manufacturer’s instructions. Complementary DNA (cDNA) was reverse-transcribed with the Omniscript Kit (Qiagen, USA) and was amplified in an Abi OneStep cycler (Applied Biosystems) using specific primer pairs for ferritin heavy and light chain, Fpn, TfR1, DMT1, and CGRPR. Peptidylprolyl isomerase A (PPIA) was used as an internal control gene. The results were quantified using the ΔΔCT method following standardization relative to the internal control gene (Livak and Schmittgen, 2001). Primers are listed in Table. 

**Table:** Table. Primer sequences used for qPCR

Gene	forward primer / reverse primer
PPIA	CCCACCGTGTTCTTCGACAT / CCAGTGCTCAGAGCACGAAA
DMT1	TGAATCGGGCCAATAAGCAGG / ATCAGCAAAGACGGACACGACAA
Fpn	AGAGCTGACCTGGCACCTTA / GGCCCAAGTCAGTGAAGGTA
Ferritin-H	TAAAGAACTGGGTGACCACGTGAC / AAGTCAGCTTAGCTCTCATCACCG
Ferritin-L	TGGCCATGGAGAAGAACCTGAATC / GCTTTCCAGGAAGTCACAGAGAT
CGRP-R	CCTTGGCGGAATAGGAGATAAG / GACAGAAGGGACCGCATAATAA

### 2.4. Immunofluorescence staining 

Cells were plated on PLL-coated round glass coverslips and allowed to attach overnight in 24-well plates. Primary antibodies (Cd11b: mouse monoclonal IgG, Abcam, UK; GFAP: mouse monoclonal IgG, Santa Cruz, USA; ferritin: rabbit polyclonal IgG, Santa Cruz, USA) were used at 1:200; and appropriate secondary antibody (goat poly-IgG to rabbit and donkey poly-IgG to mouse, Abcam, UK) conjugated with Alexa Fluor 488 were used at 1:400 dilution. Cells coated on coverslips were stained as described previously in the literature (Jeong and David, 2003). Briefly, after washes with HBSS, cells were incubated with the primary antibody for 30 min, followed by washes and incubation with the appropriate secondary antibody for 30 min. For surface antigens such as CD11b, cells were then fixed with 95% ethanol + 5% acetic acid for 15 min at 4 °C. Intracellular antigens (GFAP, ferritin) required cell permeabilization before incubation with the primary antibody (Schulz et al., 2011). CRLR/CGRPR1 polyclonal antibody conjugated with Alexa Fluor® 488 was used for CGRP receptor staining (Bioss Antibodies, USA) at 1:200 dilution. After washes in PBS, coverslips were mounted in antifade mounting medium containing DAPI (Life Technologies, ABD) and analyzed using a fluorescence microscope. 

### 2.5. ELISA 

After incubation with GTN, cell culture supernatants were collected and stored at –80 °C until the day of analysis. NO and MMP-9 levels were measured using commercial ELISA kits following manufacturer’s instructions (Oxford Biomedical Research, ABD; Shangai YL Biotech Co, China, respectively). 

### 2.6. Statistical analysis 

Statistical tests were performed using SPSS v.20. Analyses were performed by using the Kruskal–Wallis test for multiple comparisons and the Mann–Whitney *U* test for pairs for nonparametric tests and Student’s *t*-test and one-way ANOVA for parametric tests. Differences were considered significant at P < 0.05. Data was presented as mean ± SEM. 

## 3. Results

### 3.1. NO production and cell viability

**Figure 2:**
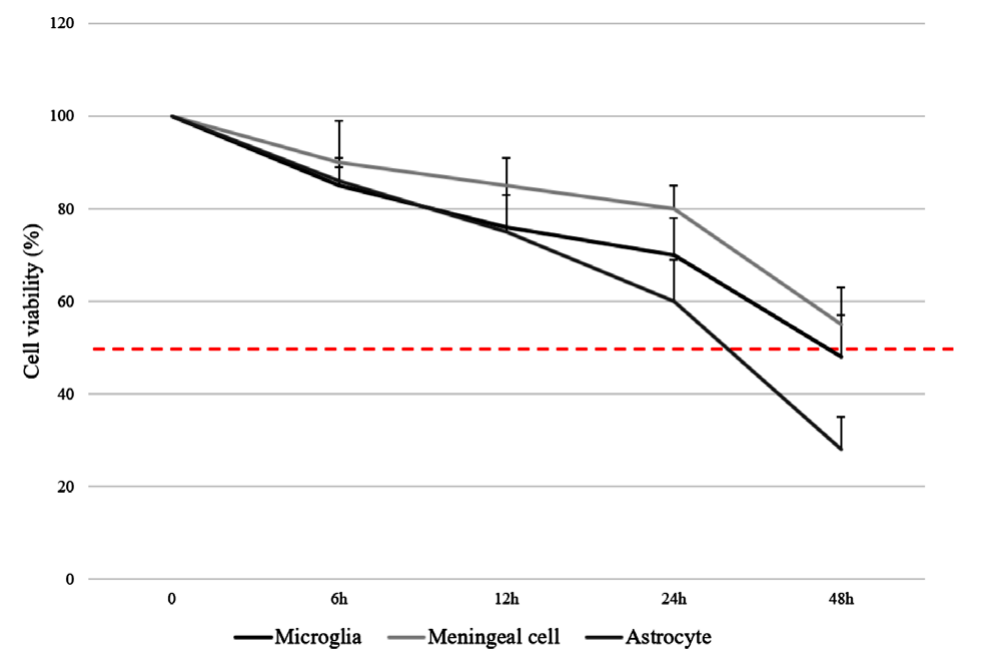
Cell viability after stimulation with 200 μM GTN in glial and meningeal cell culture (Data have been shown as mean ± SEM) (n = 3).

In our preliminary studies, we had relied on similar cell culture research in the literature and accordingly had investigated different concentrations of GTN as 100, 200, and 400 µM in cell culture for 60 min and 24 h. Two hundred µM of GTN had the prominent effect in ferritin gene expression (P < 0.05) and staining in all three cell types at 24 h (data not shown). Then we investigated the effect of 200 µM of GTN on cell viability at 6, 12, 24 and 48 h. Viable cell counts were above 50% of total cells in culture except for 48 h of incubation with GTN. Optimum conditions for incubation were accepted as 200 µM GTN for 24 h which was the highest duration of stimulation for all three cell types, for the rest of the study. Cell viabilities for glial and meningeal cells are shown in Figure 2. 

**Figure 3:**
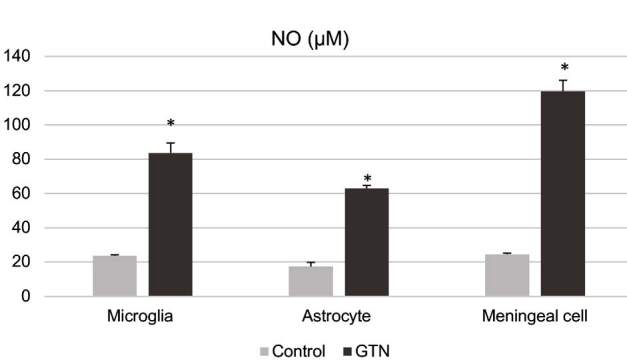
NO levels measured in microglia, astrocyte, and meningeal cell cultures after incubation with 200 μM GTN for 24 h by ELISA. Data have been shown as mean ± SEM (n = 3) (*P < 0.001).

NO levels in cell culture supernatants measured after stimulation in microglia, meningeal cells, and astrocytes are shown in Figure 3. GTN significantly elevated secreted NO levels in all three cell types (P < 0.001).

### 3.2. Iron accumulation in cells visualized by modified Perl’s histochemistry

**Figure 4:**
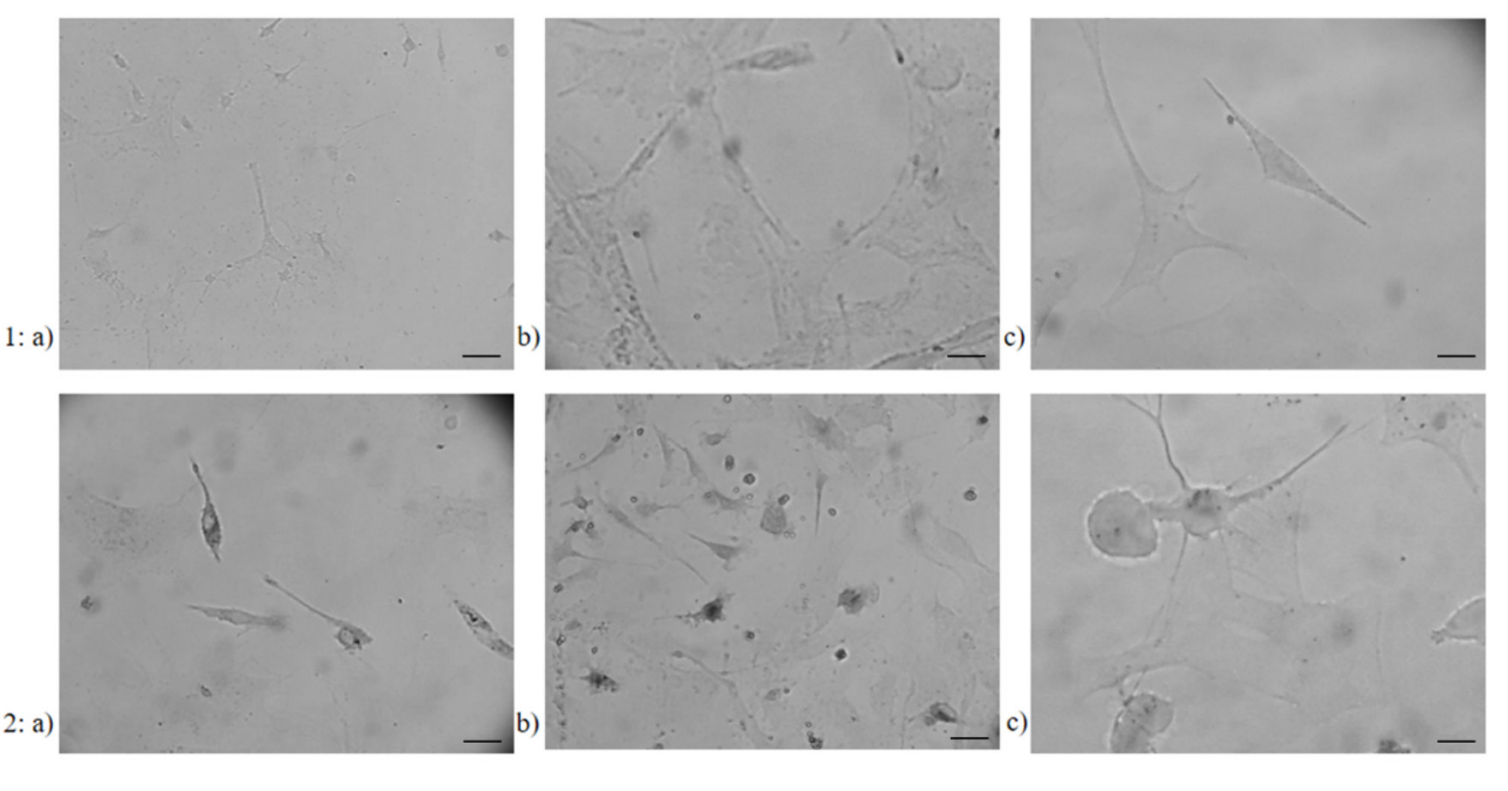
Iron accumulation showed with modified Perl’s histochemistry in 1) control and 2) GTN induced a) microglia, b) meningeal cells, and c) astrocytes (n = 3) (40× magnification, Scale bars 40 μM in a and b, 20μM in c).

Incubation with GTN increased the uptake of iron in the cell. This increase, visualized by the modified Perl’s histochemistry, was more apparent in microglia and meningeal cells (Figure 4) compared to their own controls. In astrocytes, although the number of cells receiving iron with GTN was increased, cells appeared paler when compared to other two cell types (Figure 4).

### 3.3. Expression of iron storage and trafficking proteins shown by qPCR 

**Figure 5:**
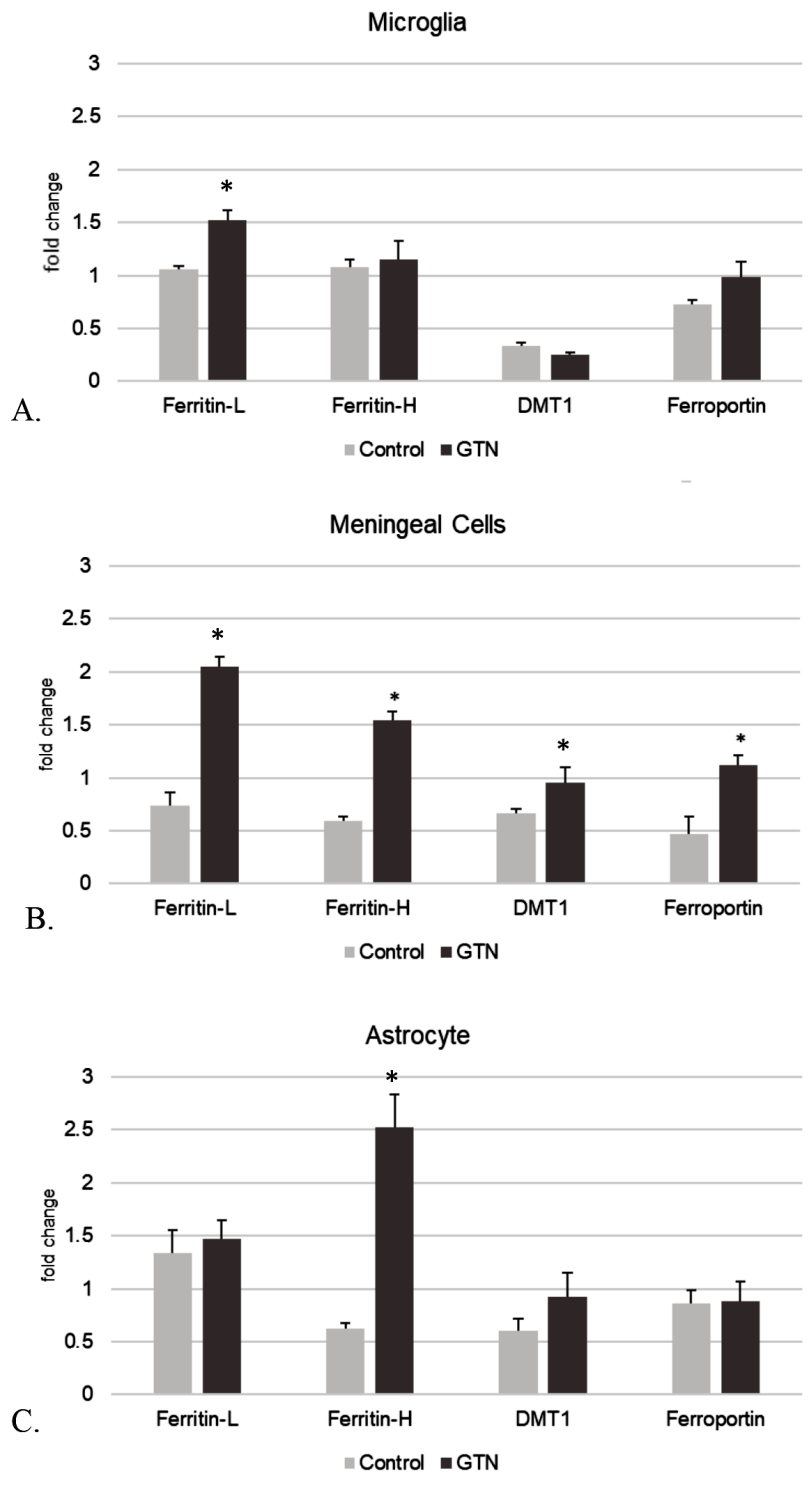
Ferritin-L, ferritin-H, DMT1, and ferroportin expression in primary cell culture of A. microglia, B. meningeal cells, and C. astrocytes after 24-h incubation with GTN (Data have been shown as mean ± SEM) (n = 3) (*P < 0.05).

**Figure 6:**
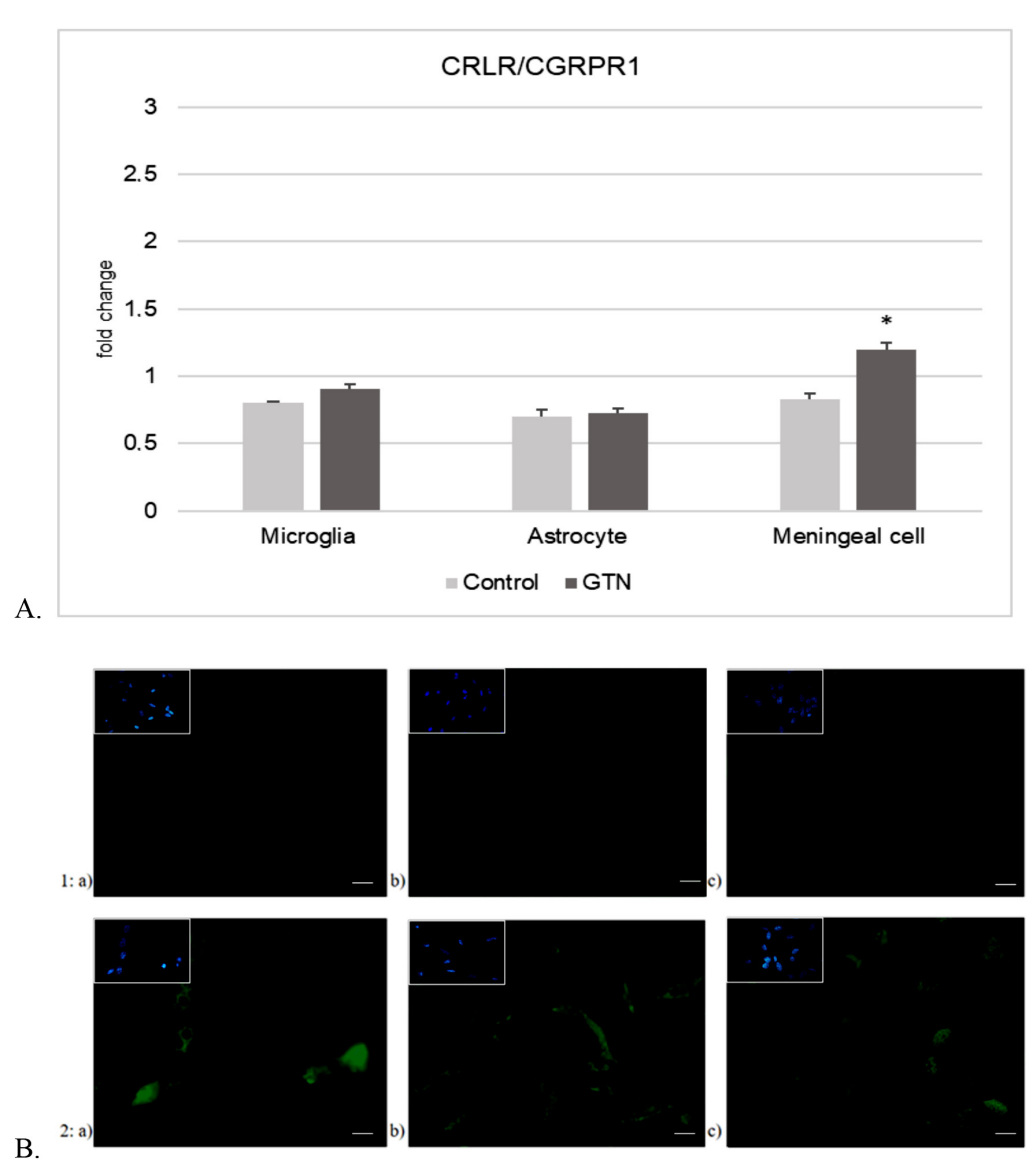
A. CRLR/CGRPR1 expression in mouse primary cell culture of microglia, meningeal cells, and astrocytes after 24-h incubation with GTN (Data have been shown as mean ± SEM) (n = 3) (P < 0.05). B. Representative fields of CRLR/CGRPR1 immunofluorescence staining in 1) control and 2) GTN induced a) microglia, b) meningeal cells, and c) astrocytes (n = 3) (40× magnification. Scale bars 40 μm). The same field for each different cell type and condition was captured with DAPI used for nuclear staining in all images.

Alterations of fold changes in expression of iron storage and transport proteins after 24-h incubation with GTN are shown in Figure 5. In microglia (Figure 5A), GTN significantly increased ferritin-L (P < 0.05) but did not affect ferritin-H and DMT1. In meningeal cells (Figure 5B), both ferritin-H and ferritin-L expressions increased significantly with GTN (P < 0.05). Ferroportin expression increased significantly together with DMT1 expression after incubation with GTN (P < 0.05). Ferritin-H expression increased after stimulation in astrocytes (P < 0.05), while ferritin-L and the transport proteins did not alter (Figure 5C). CGRP-R expression was increased after GTN stimulation significantly in meningeal cells (P < 0.05) (Figure 6A). 

### 3.4. Immunofluorescence staining of ferritin and CRLR/CGRPR1

**Figure 7:**
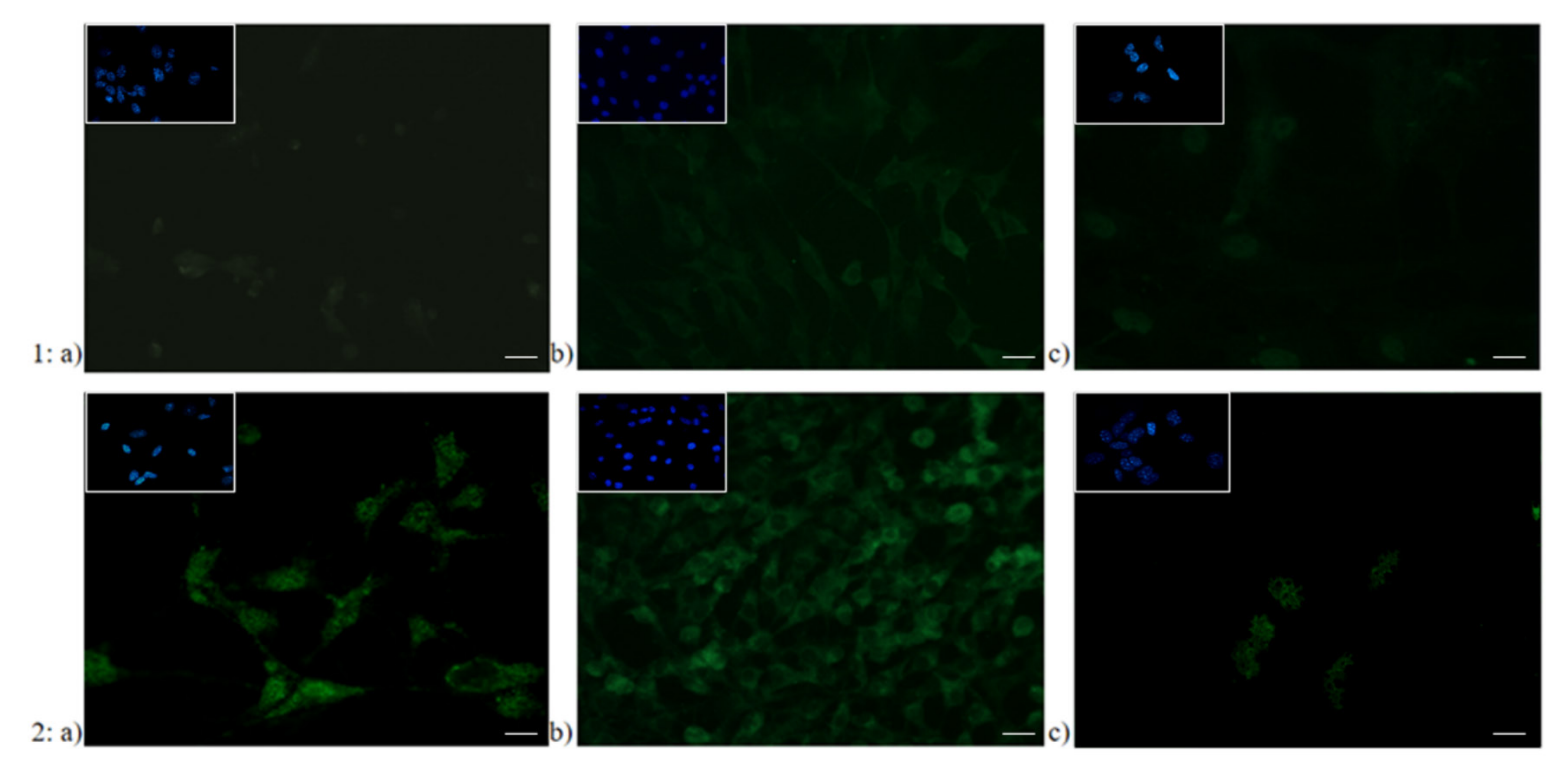
Representative fields of ferritin immunofluorescence staining in 1) control and 2) GTN induced a) microglia, b) meningeal cells, and c) astrocytes (n = 3) (40× magnification. Scale bars 40 μm). The same field for each different cell type and condition was captured with DAPI used for nuclear staining in all images.

As for relation to CGRP-R expression, immunofluorescence staining was observed in microglia and more apparently in meningeal cells (Figure 6B). It has been visually determined that ferritin stain was easily detectable in meningeal cells and microglia, where it was more apparent in meningeal cells, and indistinct in astrocytes (Figure 7). 

### 3.5. MMP-9 production 

**Figure 8:**
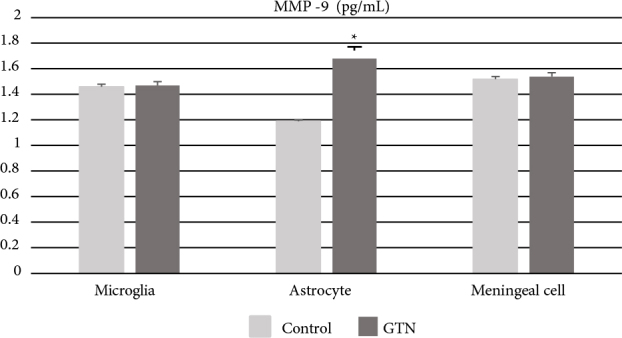
MMP-9 levels measured in microglia, astrocyte, and meningeal cell culture after incubation with 200 μM GTN for 24 h. Data have been shown as mean ± SEM (n = 3) (*P < 0.05).

GTN increased secreted MMP-9 levels in astrocytes significantly (P < 0.05) but did not cause a change in microglia or meningeal cells (P > 0.05) as measured with ELISA (Figure 8) in cell culture supernatants.

## 4. Discussion

In this study, effects of GTN stimulation on iron homeostasis in glial and meningeal cells were investigated in primary cell culture using different parameters. 

GTN is a NO donor that triggers neuropeptide release and vasodilation from trigeminal fibers (Reuter et al., 2002; Olesen 2008). Christansen et al. (1999) showed that intravenous infusion or sublingual administration of GTN induced headache shortly in healthy individuals. However, only in migraineurs, this initial phase was followed by a second phase of pain, defined as migraine-type headache which occurs after 4–6 h of infusion. Because of this feature, GTN has been identified and well confirmed to simulate migraine in both clinical and experimental research. Reuter et al. (2002) showed that an inflammatory response was triggered in dura mater in addition to iNOS expression and NO production at 4–6 h of GTN stimulation and iNOS expression increased significantly, especially in meningeal macrophages. In our study, 24-h stimulation with 200 µM of GTN did not affect cell cytotoxicity, and caused significant NO production in primary microglia, astrocyte, and meningeal cell cultures (Figure 3).

The proper homeostasis of cellular iron is important to prevent iron overload or iron starvation in cells. Although it is essential for the activity of various metabolic enzymes, excessive iron could be harmful via iron-mediated free radical damage at the molecular level. Impairment of iron homeostasis might lead to dysfunction of iron metabolism and neurodegeneration in CNS. Glial cells play a critical role in regulating iron homeostasis. A complex system of interactions involving several transport and storage proteins, located both on the glial cell surface or intracellularly, could regulate the levels of cellular iron (Zarruk et al., 2015). Zarruk et al. (2015) showed in experimental autoimmune encephalitis (EAE) that iron was accumulated in macrophage/microglia, but not in astrocytes. The difference in the expression of iron transport proteins in these two glial cell types appears to underlie the differences in its cellular accumulation.

In our study, GTN stimulation increased the iron uptake within the cell when compared to control, which was shown with modified Perl’s histochemistry, particularly in meningeal cells and microglia. In contrast, histochemical staining in astrocytes was remarkably pale (Figure 4). This suggests that the deterioration in the cellular iron homeostasis might have directly contributed to the pain process in microglia and meningeal cells, the key players of migraine pathogenesis. 

Ferritin is the primary iron storage protein in the cell. It has two different forms described as cytosolic and mitochondrial. Cytosolic form is the storage protein that stores iron in a 24-subunit monomer, consisting of heavy (-H) and light chain (-L) (Bogdan et al., 2016). Iron in ferric form (+3) is stored in ferritin in a mineralized and nontoxic form. According to Finazzi and Arosio (2014), the extent to which heavy and light chains are expressed varies according to physiological conditions and tissue type. Together with the mitochondrial form, ferritin-H catalyzes the oxidation of ferrous iron to the ferric form, which is necessary for the storage of iron in the cavity. In contrast, ferritin-L carries the necessary structures for nucleation and hydrolysis. It is important that these two chains are in a certain balance in terms of cytosolic iron storage and release. Ferritin-L is the most stable form of stored iron and it is dominantly expressed in cells located in organs such as the liver and spleen. Ferritin-H is found significantly higher in the heart and brain tissue with a high iron oxidation activity. In CNS, both forms were found in oligodendrocytes, while ferritin-L was shown to be expressed at a higher level in other glial cells (Finazzi and Arosio, 2014). Processes such as oxidative stress, inflammation, and hypoxia cause these proteins to be expressed at different rates. In addition, the increase in transcription-dependent ferritin-H in neurons was related to µ opioid receptors (Sengupta et al., 2009). 

In our study, GTN stimulated the expression of ferritin-L in microglia, ferritin-H in astrocytes, but increased both ferritin-H and ferritin-L expressions in meningeal cells (Figure 5). As mentioned above, the expression rate of iron transport proteins is one but not the only reason of this difference in accumulation of iron in different cell types. In addition to the uptake by transport proteins, another iron source of microglia is the phagocytosed iron released by cells that have been damaged within the environment. In contrast, studies showed that for example, in EAE, astrocytes encapsulated iron via different transport proteins (Zarruk et al., 2015). In our study, DMT1, which is known to be frequently expressed in glial cells and particularly associated with inflammatory processes (Zarruk et al., 2015), was chosen to evaluate the effect of GTN stimulation. DMT1 increased significantly in meningeal cells after incubation with GTN, but its expression did not change in microglia and astrocytes. This result suggests that a different influx protein such as the transmembrane Zrt- and Irt-like protein 14 (Zip14) might probably play a role for iron intake during the GTN-induced transport process in microglia. 

As also shown in our study, microglia are more prone to iron deposition than astrocytes. This suggests that both the uptake and the removal of iron are important factors regulating other metabolic functions of the cell. The major proteins that release iron out of the cell have been identified as ferroportin and ceruloplasmin. While ceruloplasmin is a specific carrier for astrocytes, ferroportin is of great importance in the removal of iron from other cells including microglia (Zarruk et al., 2015). Ferroportin is located on the cell surface and its function is controlled by various regulatory proteins. Inflammation increases iron accumulation especially in neurons and microglia by regulating DMT1 and ferroportin expressions (Urrutia et al., 2013). Zarruk et al. (2015) reported that in EAE, microglia suppressed iron excretion by internalizing ferroportin through the cell membrane. Another reason for remarkably increased iron accumulation in microglia compared to astrocytes might have been attributed to the absence of ceruloplasmin (Jeong and David, 2003; Rathore et al., 2008; Schulz et al., 2011). Therefore, microglia are defined as more sensitive cells than astrocytes in terms of iron accumulation and toxicity (Zarruk et al., 2015). In our study, ferroportin expression was significantly increased in meningeal cells after GTN stimulation, whereas no significant change was found in astrocytes and microglia (Figure 5). That iron is stored in meningeal cells as light chain suggests that the increase in ferroportin could have activated as a survival factor for the cellular iron storage. 

Astrocytes are glial cells with multiple functions in CNS. The CNS is in contact with capillaries via astrocytes’ cytoplasmic foot protrusions which are included in the structure of BBB (Nedergaard et al., 2013) and therefore could circulate excess iron directly (Zarruk et al., 2015). The impaired function of BBB may also play a role in migraine pathophysiology. MMPs are zinc-dependent enzymes which are involved in the regulation of the extracellular matrix and the maintenance of the cell-matrix relationship. It has been suggested that MMP-9 levels, which were known to peak during the 3rd day of the attack, could be used as a biomarker in migraine (Gursoy Ozdemir et al., 2004), and inhibition of MMP-9 might be an alternative target in treatment (Gupta 2009). In our study, MMP-9 levels measured in cell culture increased significantly with GTN stimulation in astrocytes (Figure 8). This increase once again, emphasized the role of astrocytes, particularly in the disruption of BBB integrity associated with the disease. However, unlike other cells, increased ferritin-H expression in astrocytes may also contribute to this process through its enzymatic activity. We reckon that further studies of ferritin response in a three-dimensional BBB cell culture model would be important for evaluating its effects on migraine pathophysiology.

Calcitonin gene-related peptide (CGRP) which is a very strong vasodilator in cerebrovascular system, is expressed in various brain regions involved in nociception. (Karsan et al., 2015). At the cellular level, CGRP increased the cAMP levels in culture medium, and stimulated immediate-early gene expression in glial cells (Morara et al., 2008). Blocking CGRP or its receptors with various agents has offered a novel therapeutic option in migraine. Careful consideration of exactly which subunit of CGRP receptors have been identified and where the target sites have been located for antimigraine action were critical for determination of the most successful treatment (Hay and Walker 2017). Since the presence of different structures that stored CGRP and different cells that expressed its receptor, the antimigraine site should have resided in areas rather than having been limited by BBB (Edvinsson, 2018). 

McLatchie et al. (1998) reported that CGRP receptor was a G protein-coupled receptor which required coexpression of the adapter protein RAMP1 to generate a specific response (CRLR/CGRPR1). This receptor protein had a broad distribution both in central and peripheral tissues, in particular the microvascular endothelium, and was suggested that the peptide–receptor system might have mediated important changes in the microvasculature under certain pathophysiological conditions (Benes et al., 2004). 

Following up with the above mentioned previous findings, we added that stimulation with GTN increased CRLR/CGRPR1 expression in meningeal cells. However, CRLR/CGRPR1 expression after GTN stimulation did not cause a significant increase in microglia and astrocytes compared to control (Figure 7). 

When associated with impulse-disrupted cellular iron homeostasis, the iron accumulated in the cell might have contributed to the exacerbation of the nociceptive pathway. This increase accompanied increased CRLR/CGRPR1 expression in meningeal cells (Figure 7). Collectively evaluated, astrocytes contributed more effectively to the migraine pathology via elevated MMP-9 for regulating BBB integrity. 

The effect of GTN, a well-known migraine triggering stimulus, on iron traffic was studied in 3 different types of cell cultures simultaneously, which we consider an added value of this study. Based on the results obtained, it might be clearly suggested that changes in the expression of iron storage and transport proteins are associated with nociceptive/antinociceptive effects. In addition, the evaluation of the effects of GTN stimulation on CGRP receptor in glial and meningeal cells, and its relationship with the cellular iron homeostasis provide important ideas in terms of different molecules and cells that should be focused on therapeutically. We are aware of the fact that our study has not determined quantitative information regarding the proteins, ferritin, and CRLR/CGRPR1 and that it must be further investigated to support our data. Keenly keeping in mind that glial cells behave interacting each other and with meningeal cells, coculture and in vivo studies would give an even more precious insight to resolve the hesitation whether they contribute to homeostatic or pathologic processes in the brain. 

The data obtained by our study provided that increased NO, which has been shown to play a role in the formation of inflammation, might regulate the cellular iron traffic; therefore, different cells contribute to migraine pathophysiology through different molecular pathways. Keeping in mind that intracellular iron homeostasis is known to play a role in many neurodegenerative diseases as well as healthy processes; it also may contribute to the pathogenesis of migraine through mislocalization of iron in the cell. Holistic and finer consideration of molecular dimensions of cellular iron storage and transport has the potential to contribute in many ways to the determination of the alternative targets in migraine treatment.
